# Multiple Sclerosis Research Evolves: A Closer Look at Deep Gray Matter, Sexual Function Rehabilitation, and T Regulatory Cells

**DOI:** 10.3390/jpm14020202

**Published:** 2024-02-12

**Authors:** Majid Ghareghani, Serge Rivest

**Affiliations:** Neuroscience Laboratory, CHU de Québec Research Centre, Department of Molecular Medicine, Faculty of Medicine, Laval University, Québec City, QC G1V 4G2, Canada

Multiple sclerosis (MS) is a chronic autoimmune condition that leads to the loss of myelin and, subsequently, neuronal damage in the central nervous system (CNS). The presence of white matter (WM) lesions has long been recognised as a key marker of MS; until recently, affected WM was considered the most important substrate to target during the neuroimaging revolution. However, it has become increasingly evident that the impacts on deep gray matter (DGM) are of critical importance and are strongly linked to a wide range of physical and cognitive difficulties associated with living with MS. Advances in magnetic resonance imaging (MRI) technologies, particularly volumetric MRI, diffusion tensor imaging (DTI), and quantitative susceptibility mapping (QSM), have enabled us to start tracking the microstructural changes within DGM.

Recent research has consistently illuminated the presence of DGM atrophy in people with MS, correlated with both physical debilitation and cognitive downturns. Key areas affected include the thalamus, caudate nucleus, globus pallidus, and putamen—all critical regions for motor control, cognitive operations, and various neurological functions. The shrinkage of these DGM regions is tied to worsening disability, suggesting its potential role as a marker for monitoring disease progression and evaluating treatment responses.

In the realm of advanced MRI, researchers have gained new insights into the depth and nature of DGM changes in MS. DTI metrics, such as fractional anisotropy (FA) and mean diffusivity (MD), have been pivotal in identifying signs of demyelination and axonal loss. Additionally, quantitative susceptibility mapping (QSM) provides a fresh perspective by quantifying iron accumulation in DGM areas, which may be indicative of ongoing neuroinflammatory activities. These imaging biomarkers have been key in enhancing our understanding of DGM pathology in MS.

A notable study by Feiyue Yin and colleagues [1] employed a machine learning lens to further investigate the microstructural shifts within the DGM of MS patients and their correlation with the clinical manifestations. By leveraging machine learning for pattern recognition and classification, this research underscores the usefulness of such algorithms in distinguishing MS patients from healthy individuals based on DGM changes and in predicting disease trajectory.

Yin and colleagues’ work not only echoes previous discoveries regarding DGM involvement in MS but also goes further by utilizing multivariate pattern analysis to pinpoint the regions most severely impacted and their link to clinical outcomes. The robust performance of their classification models, as evidenced by high area under the curve (AUC) values, highlights the effectiveness of merging various MRI metrics with machine learning techniques to differentiate MS patients from healthy counterparts and to connect DGM damage with clinical and cognitive impairments.

With regard to sexual health, sexual dysfunction emerges as a significant concern for women with MS, manifesting in various forms such as reduced libido, decreased arousal, orgasmic challenges, and discomfort or pain during intimate moments. These issues significantly weigh on the emotional and relational aspects of life, rooted in a complex interplay of neurological, psychological, and physical factors.

Particularly for those with relapsing–remitting MS (RRMS), characterized by cycles of exacerbation and remission, sexual health presents unique hurdles. The disease’s unpredictable nature can amplify the distress and anxiety surrounding sexual function, potentially worsening sexual difficulties.

Sexual distress, reflecting the emotional toll associated with sexual problems, holds equal importance in the conversation about sexual health. For women with MS, this distress may arise due to direct neurological impacts, concerns over body image, relationship dynamics, or the broader psychological challenges of chronic illness management. Addressing this distress is crucial for enhancing the quality of life and sexual contentment of individuals with MS.

In a randomized controlled trial, Zachariou et al. [2] studied the effects of pelvic floor muscle training (PFMT) on sexual function and distress among women with RR-MS. This study bifurcated 82 participants into a PFMT group and a control group, assessing their progress using the Female Sexual Function Index (FSFI) and Female Sexual Distress Scale-Revised (FSDS-R). The results of this investigation are striking, showing marked improvements in sexual function and distress for the PFMT group, except for the domain of pain.

Zachariou and colleagues’ findings contribute significantly to our understanding of sexual health interventions for women with RRMS. By showcasing the substantial benefits of supervised PFMT, this study presents a compelling case for its inclusion as a noninvasive therapeutic tool in MS care strategies.

In another vein, Dr. Borros Arneth’s review [3] thoroughly examined the role of regulatory T cells (Tregs) in MS, weaving together a decade of insights to dissect Tregs’ role in the pathogenesis and management of the disease. Arneth’s review meticulously assembles evidence to demonstrate Tregs’ crucial function in maintaining immune equilibrium and their influence on the progression of MS. However, this narrative also identifies a significant knowledge gap regarding the specific Treg subsets active in MS and their mechanisms of action, highlighting the promising yet untapped potential for research that targets these cells for therapeutic gains.

This collective body of work, from the intricate dynamics of DGM pathology and the nuanced challenges of sexual dysfunction in MS to the emerging therapeutic avenues, underscores the multifaceted nature of MS research. It highlights the continuous need for innovative approaches to better understand and address the complexities of this disease.
